# Utilization of healthcare services and renewal of health insurance membership: evidence of adverse selection in Ghana

**DOI:** 10.1186/s13561-016-0122-6

**Published:** 2016-09-13

**Authors:** Stephen Kwasi Opoku Duku, Francis Asenso-Boadi, Edward Nketiah-Amponsah, Daniel Kojo Arhinful

**Affiliations:** 1 Noguchi Memorial Institute for Medical Research, University of Ghana, Accra, Ghana; 2 Faculty of Economics and Business Administration, Free University of Amsterdam, De Boelelaan 1105, 1081HV Amsterdam, The Netherlands; 3 Ghana Health Service, Accra, Ghana; 4 National Health Insurance Authority, Accra, Ghana; 5 Department of Economics, University of Ghana, Legon, Ghana

**Keywords:** Health Insurance, Healthcare Utilization, Enrolment, Adverse Selection, Ghana

## Abstract

**Background:**

Utilization of healthcare in Ghana’s novel National Health Insurance Scheme (NHIS) has been increasing since inception with associated high claims bill which threatens the scheme’s financial sustainability. This paper investigates the presence of adverse selection by assessing the effect of healthcare utilization and frequency of use on NHIS renewal.

**Method:**

Routine enrolment and utilization data from 2008 to 2013 in two regions in Ghana was analyzed. Pearson Chi-square test was performed to test if the proportion of insured who utilize healthcare in a particular year and renew membership the following year is significantly different from those who utilize healthcare and drop-out. Logistic regressions were estimated to examine the relationship between healthcare utilization and frequency of use in previous year and NHIS renewal in current year.

**Results:**

We found evidence suggestive of the presence of adverse selection in the NHIS. Majority of insured who utilized healthcare renewed their membership whiles most of those who did not utilize healthcare dropped out. The likelihood of renewal was significantly higher for those who utilize healthcare than those who did not and also higher for those who make more health facility visits.

**Conclusion:**

The NHIS claims bill is high because high risk individuals who self-select into the scheme makes more health facility visits and creates financial sustainability problems. Policy makers should adopt pragmatic ways of enforcing mandatory enrolment so that low risk individuals remain enrolled; and sustainable ways of increasing revenue whiles ensuring that the societal objectives of the scheme are not compromised.

## Background

### Introduction

Most low and middle income countries have been facing challenges of raising revenues for financing essential healthcare services. Over the past two to three decades, many sub-Saharan African (SSA) countries have found it increasingly difficult to mobilize sufficient funds for financing health care particularly for the poor and vulnerable in society. The WHO in 2005 passed a resolution to support any of its member states, especially low income countries who develop strategies to mobilize more resources for healthcare through risk pooling to improve access to healthcare for the poor and deliver quality healthcare [1]. Some SSA countries responded to this call by focusing on social health insurance (SHI) schemes and since then interest in SHI has gained strength in SSA as shown by efforts in Kenya, Lesotho, Nigeria, Rwanda, South Africa, Swaziland, Tanzania, Uganda, Zambia, Zimbabwe and Ghana in implementing SHI schemes. Available empirical evidence indicates that these SHI schemes have provided financial protection to the poor and vulnerable in terms of reducing out-of-pocket expenditures and also improving utilization of both inpatient and outpatient care [2, 3]. Most of these SHI schemes are however facing challenges such as low enrolment coverages, poor quality of healthcare services, inefficient provider payment mechanisms, high claims bill, moral hazard and adverse selection which have the potential of eroding the gains made [2, 3]. The need to critically investigate these challenges, particularly adverse selection and find policy solutions to them, is more relevant now than ever.

In 2004, Ghana started full implementation of the National Health Insurance Scheme (NHIS). The NHIS was established with the aim of making quality healthcare accessible and affordable to all people living in Ghana, particularly the poor and vulnerable. To protect vulnerable populations, the NHIS has an exemption policy that exempts children under 18 years, elderly aged 70 years and above, Social Security and National Insurance Trust (SSNIT) contributors and pensioners, pregnant women, Indigents and Livelihood Empowerment Against Poverty (LEAP) beneficiaries from the payment of annual premium. After a decade of NHIS implementation, available statistics indicates that the scheme has made some progress towards this aim. Active membership increased from 1,348,160 in 2005 to 10,638,119 in 2009. Active membership however decreased to 8,163,714 in 2010 and increased to 8,885,757 in 2012, an increase of 8 % over the 2011 figure of 8,227,823 and representing 35 % of the Ghanaian population [4].

Out-patients visit increased over forty fold from 0.6 million visits in 2005 to 16.9 million visits in 2010 and to 25.5 million visits in 2011. It however decreased slightly to 23.9 million visits in 2012. In-patient utilization also increased more than thirty-fold from 28,906 admissions in 2005 to 724,440 admissions in 2010 and to 1,451,596 admissions in 2011. In-patient utilization however decreased to 1,428.192 admissions in 2012. These increases in out-patients and in-patients utilization rates were accompanied by increases in the claims bill over the same period. Claims bill increased from GH₵7.60 million in 2005 to GH₵616.47 million in 2012 representing 78.2 % of the total NHIS expenditure of GH₵788.32 million as against total revenue of GH₵773.83 million resulting in a net operating deficit of 14.49 million for 2012 which is financed through borrowing from banks [4]. This high claims bill that threatens the future financial sustainability of the scheme is expected to rise even further in the coming years. The National Health Insurance Authority (NHIA) attributes the high claims bill to increasing number of active members and moral hazard associated with insurance schemes. To contain the increasing claims bill, the NHIA in 2012, strengthened the clinical and Internal Audit Division, linked treatments to diagnosis, piloted a new prescription form with the intention to deploy it across the country, established Claims Processing Centers and introduced capitation as an additional provider payment mechanism [4]. These measures were put in place to reduce inefficiencies and fraud associated with claims processing and the insurance system as a whole.

Recent empirical studies have established a positive relationship between health insurance coverage and increased utilization of healthcare services. These studies indicate that health insurance reduces out-of-pocket expenditure and increases access and utilization of formal healthcare, particularly for those in the poorest wealth quintile who need healthcare most [5–7]. Although by design the NHIS is supposed to be mandatory for all people living in Ghana, in practice it is voluntary because there is no legal enforcement, making the scheme susceptible to adverse selection. The NHIS law stipulates that the premium of scheme members shall be determined by the NHIA board in consultation with the Minister of health and take into account the social nature of the scheme (Section 28 sub-section 3, NHIS Act 852) [8]. However, the premium for each district is set based on the socio-economic characteristics of that district without due consideration to the risk levels such that both high and low risk individuals are offered the same premium. Whiles formal sector employees who are SSNIT contributors are exempted from premium and therefore pay only a small registration fee to enrol, informal sector employees pay the premium plus the registration fees. Thus the premium paid by low risk individuals may exceed the actuarially fair levels for their risk status whiles that paid by high risk individuals may be lower than the fair levels for their risk status. Although the funding arrangements under the NHIS is such that all residents in Ghana who consume Value Added Tax (VAT) taxable goods and services make a 2.5 % contribution of the value of the good or service consumed to the NHIS fund, and the government also allocates funds to the NHIS fund through parliament, one of the prominent challenges of the NHIS currently is the lack of funds which has resulted in the consistent indebtedness to health care providers.

The steep increase in the out-patient and in-patient utilization rates with its associated increase in claims bills are suggestive of the presence of adverse selection in the NHIS. Adverse selection is the situation where only individuals with known underlying health conditions and high expected propensity of healthcare usage self-select into insurance schemes [9]. This paper seeks to ascertain the presence of adverse selection in the NHIS. The NHIS enrolment and utilization data from 2008 to 2013 in the Greater Accra and Western regions of Ghana was used to establish the relationship between utilization of healthcare services by NHIS subscribers and renewal of membership in subsequent years. That is, once individuals enroll, does utilization of health services have any effect on their decision to renew membership? Thus far there is paucity of studies that use actuarial data to establish the prevalence of adverse selection in Ghana’s NHIS. The paper fills this gap in the literature and also contribute to the literature on social health insurance in resource constraint countries.

The rest of the paper is organised as follows. We review empirical literature on the effect of health insurance uptake on healthcare utilisation and the problem of adverse selection. This is followed by a discussion of the conceptual framework for the paper. A description of the data and statistical methods employed for the analysis is then presented, followed by the presentation of results of the statistical analysis. The results are then discussed with some conclusions and policy recommendations.

### Literature review

Theoretically, insurance coverage is expected to affect individuals’ behavior through several distinct mechanisms. The first is what is known in the economic literature as the moral hazard problem, where insurance coverage make individuals feel safe and adopt more risky behaviors and lifestyles with associated increase in healthcare utilization. The second is when insurance coverage makes orthodox medical treatment cheaper for rational decision makers than other forms of treatment and hence changes the choices they normally would have made in the event of health problems towards increased utilization of orthodox healthcare services [9–11]. Third, is the problem of adverse selection, which is the tendency of individuals to avoid taking up health insurance unless they are sure of an underlying health problem with a high propensity of healthcare utilization. This results in the situation where only the sick end up enrolling in health insurance with its consequences on increased utilization and cost [12]. Finally, individuals would want to make use of health services as a way of recovering part of the insurance premium paid. This behavior has been well explained in models of loss aversion by Kahnemann & Tversky [13].

Empirically, several studies have been conducted in developed economies to assess the effect of insurance uptake on healthcare utilization behavior through any of the mechanisms described above [14–17]. The evidence from these studies are that health insurance coverage leads to increased healthcare utilization. In the developing world, some studies have also been conducted that provide empirical evidence of a positive relationship between insured status and increased healthcare utilization [2, 3, 16–19]. In Ghana specifically, a number of studies have been conducted that provide evidence of a positive relationship between insurance coverage and increased healthcare utilization [7, 11, 20]. The missing link however in the Ghanaian context is whether increased healthcare use once enrolled has an effect on the decision to renew enrolment and suggestive of the presence of adverse selection.

Adverse selection has been extensively studied in developed countries with a focus on employer and government insurance schemes [9, 21–26]. In the developing world, the phenomenon of adverse selection has been studied in greater detail with mixed findings. Whiles some of these studies [25, 27, 28] found evidence of the presence of adverse selection, others [28–31] found no evidence of the presence of adverse selection in the schemes they investigated. In Ghana, since the implementation of the NHIS in 2005, to the best of our knowledge only two studies have examined the problem of adverse selection. Rajkotia & Frick [32] tested for the presence of adverse selection in the NHIS in Nkoronza district in the Brong Ahafo Region in Ghana after the district scheme introduced a premium waiver for all children less than 18 years in exchange for full household enrolment as a way of discouraging households from selectively enrolling their sickest (high-risk) members. They found that household enrolment cost was positively correlated with the number of children in the household and the odds and intensity of outpatient use by children. They concluded that child premium waiver is an important incentive for household enrolment and provides evidence suggestive that adverse selection has effectively been contained but not eliminated. Another study by Amponsah [33] tested for the presence of adverse selection, moral hazard, and income effect in the NHIS. He found that after controlling for selection bias, the likelihood of purchasing health insurance increases with health risk which is evident of adverse selection.

Although these studies provide empirical evidence of the presence of adverse selection in health insurance in Ghana, they used datasets that spans one and two periods of time respectively. Whiles Rajkotia & Frick [32] used the enrolment and claims database of Nkoronsa district NHIS from August 2005 to July 2006, Amponsah [33] used the Ghana Living Standards Survey (GLSS) waves IV and V, conducted by the Ghana Statistical Service (GSS) in 1998/99 and 2005/06. This study however, uses routine enrolment and utilization database of the NHIS from two regions in Ghana that spans a period of 6 years from 2008 to 2013 to assess healthcare utilization by insured clients and its effect on renewal of insurance membership and ascertain the presence of adverse selection in the NHIS.

### Conceptual framework

Rational, risk averse individuals are expected to enrol in the NHIS to protect themselves from catastrophic health expenditure in the event of ill health. However, once enrolled, there is the likelihood for high risk individuals to utilise health services more than low risk individuals due to the underlying poor health conditions. Over time, low risk individuals who enrol but make little or no use of health services may drop out of the insurance scheme because they feel they are not benefiting from the premium paid, leaving only high risk individuals in the insurance pool which may lead to the problem of adverse selection and its associated high claims bill. The expectation is that healthcare utilisation of insured in a particular year will be positively associated with the renewal decision in the following year. Thus enrolled individuals who utilize healthcare in previous year will renew their membership in current year if adverse selection is present in the NHIS.

**Fig. 1:**
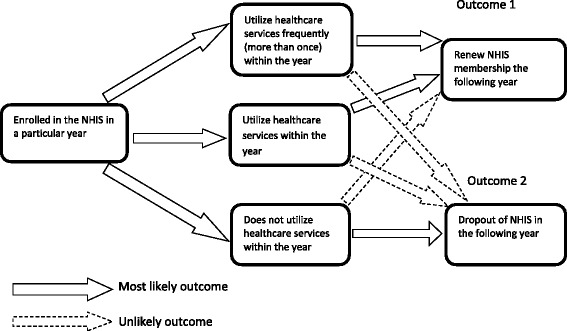
Conceptual framework of effect of healthcare utilization on Insurance renewal

Willingness to renew NHIS membership depends on several factors one of which is the prior knowledge of health status. Health status will influence NHIS renewal such that those in good health are unlikely to renew whiles those of poor health are most likely to renew. Healthcare utilization was used as a proxy to measure health status, where a health facility visit in a particular year for curative services of which the NHIS reimburses the healthcare provider is taken as relatively poor health status and no health facility visit is taken as relatively good health status [32]. Secondly, the intensity of health services usage once enrolled will influence NHIS renewal such that those who visited a health facility once (high-risk) or more than once (very high-risk) are more likely to renew their membership whiles those who did not visit a health facility (low-risk) may drop out [32]. The effect of healthcare utilization on the renewal decision is illustrated in Fig. 1.

From Fig. 1, we hypothesize that enrolling in the NHIS and utilizing healthcare (at least once a year) would most likely lead to renewal of NHIS membership in the following year (Outcome 1). We further hypothesize that enrolling in the NHIS and not utilizing healthcare in that year would most likely lead to drop out of the NHIS. The presence of adverse selection will be evident if over time, the same individuals who enroll and utilize healthcare renew their membership.

## Methods

### Data

This study is part of a larger research, called the Client-Oriented Health Insurance System in Ghana (COHEiSION) Project which is funded by the Netherlands Ministry of Foreign Affairs and the Science for Global Development (WOTRO), under the Global Health Policy and Systems Research (GHPHSR) programme. The study was conducted in the Greater Accra and Western regions in Ghana. The Greater Accra Region with the capital city of Ghana, Accra, has a largely urban population of about 4 million accounting for 16.3 % of the national population. The Western region has a predominantly rural population of about 2 million representing 9.6 % of the national population [34]. These two regions provided the rural/urban balance for the Project in terms of population and socio-economic activities. Only the NHIS enrolment and utilization data for Greater Accra and Western regions was released to the COHEiSION Project for this paper because the NHIA data availability policy does not allow the release of patient level data of more than two regions to third parties.

The enrolment data consisted of individuals enrolled in the NHIS in these two regions from 2008 to 2013, and the utilization data consisted of NHIS subscribers who visited accredited health facilities at least once in these two regions from 2008 to 2012. These two data sets contained information on unique NHIS member identification (ID) number, name of subscriber, date of birth, sex, scheme code, community code, community name, phone number, first enrolment date, enrolment end date, premium exemption category, dates of healthcare service usage, total cost per healthcare usage and utilized services claims date.

Stata version 12.1 was used to set the data up for analysis. The enrolment and utilization data were separated into the specific years (2008, 2009, 2010, 2011, 2012 and 2013). The enrolment data was then cleaned by dropping all duplicate observations for the specific years since no individual is expected to register more than once in a specific year in the NHIS data. Thus for each year, using the NHIS ID number as the unique identifier, any individual whose NHIS ID and name appears more than once, was treated as a duplicated registration. The duplicates were therefore dropped leaving only one NHIS ID and name for that individual in the data. For the Utilization data, since an individual can make more than one health facility visit in a particular year, it contained a lot of duplicate observations. A utilization rate variable that counted the number of times an individual visited a health facility in a particular year was generated. After generating this variable, all duplicate observations were dropped to obtain the number of individuals who visited health facilities in a particular year with their corresponding rates of utilization captured by the utilization rate variable.

The enrolment data of a particular year was then merged with the utilization data of the same year for all the years using their unique NHIS membership identification numbers. Individuals in the enrolment data that matched with their membership identification numbers in the utilization data constituted those who visited health facilities in that particular year and those that did not match constituted those who were insured but did not make any health facility visit. A utilization variable was generated using the match code obtained from the stata merging command. This dichotomous variable was assigned a value of 1 if the individual visited a health facility in that year and 0 if otherwise. To obtain the NHIS renewal in subsequent years, the data obtained after merging the enrolment and utilization data for a particular year was merged with the enrolment data of the following year (e.g. merged 2008 enrolment and utilization data will be merged with 2009 enrolment data). This was done for all the years in the data set. A complete match was not obtained since not all insured individuals in a particular year renewed their membership the following year. Individuals in a particular year whose membership numbers matched their membership numbers in the following year’s enrolment data constituted renewals and those that did not match constituted those who did not renew. A renewal variable was then generated using the match code obtained from the stata merging command. This dichotomous variable was equal to 1 if the individual renewed membership and 0 if otherwise. Other variables in the data such as sex and region of residence were also dichotomized before analysis.

### Statistical analysis

The percentage of NHIS enrolment over the 6 years period was assessed for males and females; for five age groups (children under one year, children 1–17 years, adults 18–39, ageing adults 40–59 years and the elderly above 60 years) and for the different enrolment categories to understand the pattern of enrolment among the premium paying members and dependents for the different age groups and among exempted categories. The proportion of insured utilizing healthcare services over the same period was also examined. Pearson chi-square test was performed to test if the proportion of insured utilizing healthcare at different frequencies in a particular year and renewing membership in the following year is significantly different from those utilizing healthcare at different frequencies and dropping out.

Finally, logistic regressions were estimated to examine the relationship between healthcare utilization in previous year and NHIS renewal in current year. Two components of healthcare utilization which are health facility visit irrespective of the number; and the number of health facility visits, conditioned on a particular year’s utilization were used. The first logistic regression estimation assessed the relationship between NHIS renewal in current year and health facility visit in previous year. The second logistic regression estimation assessed the relationship between NHIS renewal in current year and the number of healthcare facility visits in the previous year. These estimations were run for all the years. A positive relationship in both estimations will suggest the presence of adverse selection in the NHIS. Thus individuals who utilize healthcare services most are more likely to renew their NHIS membership in subsequent years.

## Results

### Demographic characteristics and NHIS enrolment

Table 1 summarizes the demographic characteristics and NHIS enrolment of subscribers. Out of the total number of enrollees in the two regions, the percentage living in the Greater Accra region was higher (more than 53 %) than the percentage living in the Western region (less than 43 %) for all the years (2008–2013). The NHIS has a relatively youthful membership (least mean age of 24 years for 2008 & 2013) with a higher proportion of them (least 58 % for 2008) being females in all the years. NHIS coverage for the two regions saw a steady increase from 939,559 members in 2008 to 2,207,459 in 2013 representing a 2.35 fold increase of the 2008 figure as compared to the national coverage that saw an increase from 9,914,256 members in 2008 to 10,145,196 members in 2013 representing a 1.02 fold increase of the 2008 figure. The percentage of insured renewing their membership in the two regions increased from 16.11 % in 2009 to 40.13 % in 2013 as compared to the national estimates which increased from a 43.65 % renewals in 2010 to 75.41 % renewals in 2013 [35–37]. In terms of age strata, children between the ages of 1–17 and young adults between the ages of 18–39 formed the majority of enrollees for all the years in the two regions. For enrolment categories, children under 18 years exemption category (least 37 for 2009 and highest 45 % for 2012) formed the majority of enrollees in all the years over the 6 year (2008–2013) period. This is quite similar to the national estimates where the children in the under 18 years exemptions category have always constituted the highest proportion of enrollees from 49.4 % in 2009 to 46.5 % in 2013 [35–37].

**Table 1:** Demographic characteristics and NHIS enrolment

	Year					
Characteristics	2008 (*N* = 939559)	2009 (*N* = 1045072)	2010 (*N* = 1384588)	2011 (*N* = 1753000)	2012 (*N* = 2079141)	2013 (*N* = 2207459)
Demographic						
Females (%)	57.82	58.68	58.96	59.04	59.05	58.24
Mean Age (years)	24.10 (19.73)	27.07 (20.04)	26.31 (20.35)	25.53 (20.19)	24.74 (20.20)	24.04 (20.06)
Greater Accra Region (%)	57.38	60.71	53.84	58.38	57.87	57.06
Western Region (%)	42.62	39.29	46.16	41.62	42.13	42.94
NHIS Enrolment						
Total Number Insured	939559	1045072	1384588	1753000	2079141	2207459
Number New Registrants		876655	1007219	1120036	1267786	1321657
Number Renewals		168417	377369	632964	811355	885802
Percentage New Registrants		83.89	72.75	63.89	60.98	59.87
Percentage Renewals		16.11	27.25	36.11	39.02	40.13
NHIS Enrolment by Age						
Babies Under One year (%)	4.53	0.80	0.98	1.36	2.02	2.20
Children 1–17 years (%)	39.66	38.65	41.40	42.32	43.45	46.00
Adults 18–39 years (%)	29.78	36.36	33.65	33.64	32.45	30.86
Ageing Adults 40–59 years (%)	13.08	15.70	15.55	14.91	14.45	13.91
Elderly Above 60 year (%)	12.95	8.48	8.42	7.76	7.63	7.03
NHIS Enrolment by Category						
Informal Employees (%)	33.29	35.33	36.47	37.77	36.81	32.96
Children under 18 Years (%)	42.02	37.49	40.88	42.37	44.61	43.52
70 Years & Above (%)	4.05	4.89	4.68	4.03	3.58	3.14
SSNIT Contributors (%)	15.29	12.95	11.48	10.55	9.64	9.97
SSNIT Pensioner (%)	0.52	0.52	0.43	0.35	0.29	0.26
Indigent (%)	4.61	8.47	5.60	4.30	4.39	9.62
Pregnant Woman (%)	0.20	0.33	0.44	0.61	0.66	0.51
LEAP (%)	0.10	0.01	0.02	0.01	0.02	0.02

Source: NHIA Enrolment Data from 2008 – 2013. Note: Standard deviation in parenthesis

### Healthcare utilization

**Fig. 2:**
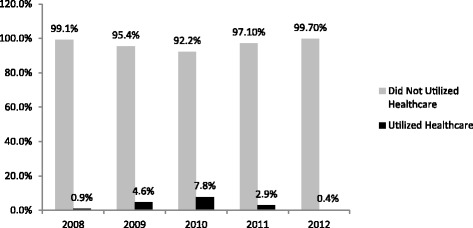
Percentage of Enrolled Utilizing Services per Year

Healthcare utilization pattern of the insured are summarized in Table 2 and Fig. 2. Health facility visits increased from 12,270 in 2008 involving 8,261 individuals to 168,602 in 2010 involving 108,124 individuals. The utilization rate however declined to 75,633 visits in 2011 involving 50,731 individuals and dropped even further to 8,870 visits in 2012 involving 7,476 individuals (Table 2). The percentage of insured utilizing healthcare also increased from 0.9 % in 2008 to 7.8 % in 2010. It then dropped to 2.9 in 2011 and dropped even further to 0.4 % in 2012 (Fig. 2).

**Table 2:** Healthcare utilization

	Year									
	2008		2009		2010		2011		2012	
	Enrolment	Utilization	Enrolment	Utilization	Enrolment	Utilization	Enrolment	Utilization	Enrolment	Utilization
Utilization										
Total Number Insured	939559		1045072		1384588		1753000		2079141	
Total Count of Service Utilization		12270		66089		168602		75633		8870
Number of Individuals Utilizing Services		8261		48431		108124		50731		7476
Percentage of Insured Utilizing Services		0.88		4.63		7.81		2.89		0.35
Frequency of Health Services Utilization										
Percentage with one visit		89.30		79.07		70.00		74.62		84.83
Percentage with two visits		8.84		15.11		18.06		15.05		12.32
Percentage with three visits		1.45		3.89		6.06		4.96		2.33
Percentage with four visits		0.29		1.23		2.64		2.18		0.43
Percentage with five visits		0.11		0.42		1.33		1.19		0.08
Percentage with more than five visits		0.01		0.29		1.92		1.99		0.01
Mean annual number of visits		1.13 (0.42)		1.30 (0.71)		1.56 (1.20)		1.49 (1.17)		1.19 (0.49)
Mean annual cost of Utilization(GHC)		16.74 (37.66)		18.83 (52.57)		17.56 (31.52)		13.84 (19.15)		17.04 (26.17)

Source: NHIA Utilization Data from 2008 – 2012. Note: Standard deviation in parenthesis

In terms of frequency of healthcare utilization, the mean annual number of health facility visits was less than 2 in all the years. The year 2008 recorded the lowest of 1.13 with a standard deviation of 0.42 visits whiles the highest of 1.56 with a standard deviation of 1.20 visits was recorded in 2010. Majority of insured (lowest 70 in 2010 and highest 89 % in 2008) who utilized healthcare in each year made only one health facility visit whiles a small proportion(least 0.01 in 2008 and 2012 and highest 1.99 % in 2011) made more than 5 health facility visits. The lowest mean annual cost of health facility visit was GH₵13.84 ($8.36) in 2011 with a standard deviation of GH₵19.15 ($11.56) and the highest of GH₵18.83 ($4.92) in 2009 with a standard deviation of GH₵52.57 ($36.21) (Table 2).

### Effect of healthcare utilization on NHIS renewals

Table 3 presents the results of Pearson chi-square test on healthcare utilization and frequency of use and NHIS renewals. Generally, the proportion of insured that utilized healthcare and renewed NHIS membership (lowest 43 in 2009 and highest 60 % in 2010) was found to be statistically significantly (*p*-value = 0.000) higher than the proportion that did not utilize healthcare (lowest 18 in 2009 and highest 46 % in 2012) and renewed their membership. Similarly, the proportion of insured that did not utilize healthcare and dropped-out of the NHIS (lowest 54 in 2012 and highest 82 % in 2009) was also found to be statistically significantly higher than the proportion that utilized healthcare and dropped-out (lowest 40 in 2010 and highest 57 % in 2009). The proportion of insured that utilized healthcare and renewed their NHIS membership increases with increasing frequency of health facility visits. A higher proportion of those that made 4 health facility visits renewed their membership than those that made 2 health facility visits. For those who made four health facility visits, 63, 74, 60, 60 and 34 % renewed their NHIS membership in the years 2009, 2010, 2011 and 2012 respectively as compared to 44, 62, 57 56 and 46 % for those who made two health facility visits in the years 2009, 2010, 2011 and 2012 respectively and renewed their NHIS membership.

**Table 3:** Healthcare utilization and NHIS renewals

	NHIS renewal status														
	2009 (*N* = 943350)			2010 (*N* = 1059921)			2011 (*N* = 1411972)			2012 (1767073)			2013 (*N* = 2081945)		
Previous Years Utilization	Dropout (%)	Renew (%)	*P*-Val.	Dropout (%)	Renew (%)	*P*-Val.	Dropout (%)	Renew (%)	*P*-Val.	Dropout (%)	Renew (%)	*P*-Val.	Dropout (%)	Renew (%)	*P*-Val.
Utilization															
Utilized Service	57.20	42.80	***	39.89	60.11	***	43.34	56.66	***	42.59	57.41	***	49.29	50.71	***
Did Not Utilize	82.13	17.87		64.71	35.29		55.23	44.77		54.05	45.95		57.43	42.57	
Total	81.91	18.09		63.58	36.42		54.32	45.68		53.73	46.27		57.40	42.60	
Frequency															
No Visit	82.13	17.87	***	64.71	35.29	***	55.23	44.77	***	54.05	45.95	***	57.43	42.57	***
one visit	57.35	42.65		40.99	59.01		43.88	56.12		42.80	57.20		48.27	51.73	
two visits	55.75	44.25		37.56	62.44		42.77	57.23		43.70	56.30		53.64	46.36	
three visits	60.00	40.30		33.62	66.38		42.19	57.81		41.99	58.01		59.77	40.23	
four visits	37.50	62.50		25.92	74.08		40.29	59.71		39.91	60.09		65.63	34.38	
five visits	55.56	44.44		27.23	72.77		40.93	59.07		37.02	62.98		66.67	33.33	
> five visits	100.00	0.00		22.86	77.14		38.55	61.45		34.09	65.91		100.00	0.00	

Source: NHIA Enrolment Data from 2008 – 2013; NHIA Utilization Data from 2008 – 2012; Note: Absolute figures in parenthesis; *** *p* < 0.001

For the years 2009 and 2013, there was 100 % drop out respectively for those who made more than 5 health facility visits. However, a close examination of the absolute figures indicates that only single individuals were involved respectively. These individuals might have died from chronic medical conditions that compelled them to make such high health facility visits and were therefore not alive to renew their membership. The NHIS routine data does not capture such drop-outs due to death.

Table 4 presents the logistic regression estimates of the effect of health facility visit on NHIS renewal. Generally, the insured who visited a health facility in a particular year were found to be significantly more likely (odds ratio = 1.66, 2.62, 1.83, 1.91, & 2.08 for the years 2009, 2010, 2011, 2012 & 2013 respectively) to renew their NHIS membership the following year than those who did not visit a health facility. Females, children under 1 year, the 70+ year exemption group, SSNIT pensioners, SSNIT contributors, pregnant women and people living in the Greater Accra region were also found to be more likely at varying extents to renew NHIS membership. All these findings were statistically significant at the 95 % confidence level.

**Table 4:** Effect of previous year’s healthcare utilization on NHIS renewal

		Year				
		2009	2010	2011	2012	2013
		Odds	Odds	Odds	Odds	Odds
Healthcare Utilization	Utilized Healthcare in Previous Year	1.66***	2.62***	1.83***	1.91***	2.08***
Sex	Male	0.99	0.92***	0.95***	0.92***	0.92***
Age Group	Age1–17 Years	0.99	0.89***	0.86***	1.11***	1.18***
	Age 18–39	0.53***	0.26***	0.34***	0.56***	0.43***
	Age 40–59	0.69***	0.44***	0.59***	0.98	0.77***
	Age 60+	0.19***	0.55***	0.73***	1.28***	0.85***
Enrolment Categories	Children under 18 Years	0.53***	0.47***	0.70***	0.99	0.74***
	70 Years & Above	5.11***	1.28***	1.24***	1.29***	1.56***
	SSNIT Contributor	1.24***	1.59***	1.59***	1.65***	1.73***
	SSNIT Pensioner	5.43***	1.67***	2.25***	2.22***	2.80***
	Indigent	0.67***	0.27***	0.44***	0.62***	0.94***
	Pregnant Woman	1.68***	1.88***	1.68***	1.44***	1.32***
	LEAP	0.34**	1.65**	1.02	1.33*	1.15
Region	Greater Accra	1.32***	0.87***	1.09***	0.83***	0.62***
	Constant	0.37***	1.67***	1.54***	1.09***	1.39***
	Number of observations	834487	997544	1298711	1644807	1965649

Source: NHIS Enrolment and Utilisation Data for the years 2008 – 2013

Note: Odds ratio are reported with reference Categories; Healthcare Utilization (Did not utilized healthcare in previous year); Sex (Female); Age Group (Babies under 1 Year); Enrolment Categories (Informal Sector Employees). Note: * *p* < 0.05; ** *p* < 0.01; *** *p* < 0.001

Finally, Table 5 presents the logistic regression estimates of the effect of frequency of health facility visits on NHIS renewal. We found that those who utilized healthcare most are more likely to renew their NHIS membership than those who do not utilize healthcare. Increases in the frequency of healthcare usage were accompanied by corresponding statistically significant increases in the likelihood of NHIS renewal for the years 2010, 2011 and 2012. However, only one visit (1.68) and two visits (1.61) for 2009; and for 2013 only one visit (2.14), two visits (1.76) and three visits (1.65) respectively had statistically significant likelihood of renewal in the NHIS (see Table 5).

**Table 5:** Effect of number of health facility visits on NHIS renewal

		Years				
		2009	2010	2011	2012	2013
		Odds	Odds	Odds	Odds	Odds
Healthcare Utilization	One Visit	1.68***	2.48***	1.82***	1.92***	2.14***
	Two visits	1.61***	2.90***	1.86***	1.86***	1.76***
	Three Visits	1.33	3.51***	1.88***	1.86***	1.65**
	Four Visits	2.76	4.67***	1.93***	1.95***	0.70
	Five Visits	1.22	4.48***	1.96***	1.98***	0.72
	More than Five Visits		6.76***	1.72***	2.08***	
Sex	Male	0.99	0.92***	0.95***	0.92***	0.92***
Age Group	Children 1–17 Years	0.99	0.89***	0.86***	1.11***	1.18***
	Age 18–39	0.53***	0.26***	0.34***	0.56***	0.43***
	Age 40–59	0.69***	0.44***	0.59***	0.98	0.77***
	Age 60+	0.19***	0.55***	0.73***	1.28***	0.85***
Exemption Categories	Children under 18 Years	0.53***	0.47***	0.70***	0.99	0.74***
	70 Years & Above	5.11***	1.28***	1.24***	1.29***	1.56***
	SSNIT Contributor	1.24***	1.59***	1.59***	1.65***	1.73***
	SSNIT Pensioner	5.43***	1.67***	2.25***	2.22***	2.80***
	Indigent	0.67***	0.27***	0.44***	0.62***	0.94***
	Pregnant Woman	1.68***	1.88***	1.68***	1.44***	1.32***
	LEAP	0.33**	1.65**	1.02***	1.33*	1.15
Region	Greater Accra	1.31***	0.87***	1.09***	0.83***	0.62***
	Constant	0.37***	1.67***	1.54***	1.09***	1.39***
	Number of observations	834487	997544	1298711	1644807	1965648

Source: NHIS Enrolment and Utilisation Data for the years 2008 – 2013

Note: Odds ratio are reported with reference Categories, Healthcare Utilization (Did not utilized healthcare in previous year); Sex (Female); Age Group (Babies under 1 Year); Enrolment Categories (Informal Sector Employees). Note: * *p* < 0.05; ** *p* < 0.01; *** *p* < 0.000

## Discussion

The results from the enrolment and utilization pattern suggest that just as the enrolment coverage has been increasing over the years since the inception of the NHIS, so has the total count of health facility visits as well as the number of individuals making those visits. However, the proportion of insured who utilized healthcare in any given year over the 6 years period never exceeded 8 %. If this is the case, why then is the NHIS claims bill outstripping its revenue? According to the NHIS, the claims bill increased from 183.01 million Ghana Cedis in 2008 to 548.71 million Ghana Cedis in 2011 representing 88.82 % of total revenue from all sources for 2011 and increased even further in 2012 to 616.47 million Ghana Cedis representing 79.66 % of the total NHIS revenue from all sources for that year [37]. This means that although a very small proportion of the insured in any given year utilize healthcare, the claims bill from this utilization is always in excess of 80 % of NHIS revenue. While acknowledging that excessive utilization by the insured is a cost-driver, it is also obvious that false claims and fraud perpetuated by some NHIS staff in collusion with healthcare providers as reported by the NHIS [4] is also to blame for the high unsustainable claims bill which threatens the future of the scheme.

The chi-square test results also suggest that over the years, majority of insured who do not utilize healthcare drop out of the scheme whiles those who utilize healthcare renew their membership. An even higher proportion of the insured who makes more health facility visits in any particular year renew their membership as compared to those who do not utilize healthcare in that year. Again the likelihood of renewal was higher for those who utilize healthcare than those who do not and also higher for those who make more health facility visits than those who do not. This is suggestive of the presence of adverse selection in the NHIS. This finding is in line with Cutler’s assertion that “almost all health insurance systems where individuals are allowed choice of insurance have experienced adverse selection” [38]. The possible explanation for this phenomenon is that once people enroll and utilize healthcare, those with underlying medical conditions (high risk individuals) become aware of their conditions and the need for future healthcare consumption. Even those who are not insured but realize they have chronic conditions and cannot afford out-of-pocket payment for their frequent healthcare consumption, enroll in the NHIS with the expectation that they will be able to access healthcare with their NHIS card at the next visit within the 3 months waiting period. Since the NHIS annual premium is set without consideration to health risk levels, high risk and low risk individuals pay the same amount of premium. This makes it expedient for high risk individuals with knowledge of their underlying health conditions to enroll/renew their membership to avoid paying out-of-pocket the high cost of healthcare any time they visit a health facility. On the other hand, low risk individuals who do not utilize healthcare feel they are not making use of the premium paid and therefore drop out of the NHIS thereby creating a situation where most of those who utilize healthcare renew their membership whiles those who do not utilize healthcare drop-out.

These findings are consistent with findings by Rajkotia & Frick [32] and Amponsah [33] that provided evidence of the presence of adverse selection in the NHIS. It is further in line with the conclusion by Rajkotia & Frick [32] that the design of the NHIS to offer premium exemptions to children, the aged 70+ years, pregnant women and vulnerable groups such as indigents and LEAP beneficiaries in a way has largely contained but not eliminated adverse selection. The granting of exemptions was partly to contain adverse selection by ensuring that both high and low risk individuals are enrolled. There is evidence that adverse selection can be contained this way and has been demonstrated in a study in micro insurance in China by Wang et al. [27]. They found that offering household enrolment reduces adverse selection by bringing into the insurance pool those low risk members of the household who would not otherwise enroll if the insurance was offered at the individual level. However, this aim of containing adverse selection in the NHIS has not been fully achieved because although by design the NHIS is mandatory for all people living in Ghana, in practice it is voluntary because there is no legal enforcement, making it possible for low risk individuals to opt out living high risk individuals mostly from the exemption groups in the insurance pool. Other studies from community-based health insurance schemes in Africa by Atim [39] and Diop [40] also pointed to similar conclusions.

Although the NHIA in recent times have consistently expressed concern about the ever increasing claims bill and its effect on the future financial sustainability of the scheme, there has been very little research to date on NHIS enrolment with a representative data to ascertain whether utilization of healthcare by the insured has any effect on renewals and whether the problem of adverse selection exists in the NHIS. This paper provides evidence, suggestive of the presence of adverse selection in the NHIS. There is therefore the need for policy makers to put in place measures to enforce the mandatory enrolment by all people living in Ghana so as to contain adverse selection.

The analyses contained in this paper are however subject to the following limitations in the data. The paper assumes that enrolment in the NHIS begins in January of any particular year and ends in December of that same year. Utilization of healthcare in a particular year was therefore taken as any health facility visit from the 1st January to 31st December of the same year. However, in reality this may not be the case since an individual who enrolls in the NHIS in May 2008 will have membership valid up until April 2009 and will have to renew his membership in May 2009. For the purposes of the analysis in this paper, this individual is deemed to have enrolled in 2008, but if he/she does not visit a health facility between May 2008 and December 2008 but visits a health facility in February 2009, it is assumed he did not utilize healthcare during the 2008 enrolment period. If he/she renews his membership in May 2009 and does not utilize healthcare for the rest of 2009, his February 2009 health facility visit is taken as healthcare utilization for the 2009 enrolment period. Any inferences made from the findings in this paper should therefore be done with caution taking into consideration the above limitation. Nevertheless, these limitations by no means invalidate the findings from this paper.

## Conclusion

Our analysis of the effect of previous utilization of healthcare and frequency of usage by the insured on the decision to renew membership using NHIS routine data (2008 – 2013) provides evidence of the presence of adverse selection in Ghana’s NHIS. Only a very small proportion of the insured in any given year utilize healthcare. The proportion of the insured that makes more health facility visits in any particular year and renew their membership was also found to be higher than those who do not utilize healthcare and renew their membership. The likelihood of renewal was found to be higher for those who utilize healthcare than those who do not and also higher for those who make more health facility visits than those who do not.

The existence of adverse selection in the NHIS has the potential of creating financial sustainability problems since high risk individuals who self-select into the scheme demand more healthcare services and hence increases the claims bill. Policy makers should adopt more pragmatic ways of encouraging low risk individuals to remain enrolled in the NHIS by enforcing the mandatory enrolment and also find sustainable ways of increasing the revenue base of the scheme. We suggest that effective mechanisms are put in place to obtain accurate data on income levels of Ghanaians particularly those in the informal sector of employment so that NHIS premium levels can be set based on income levels. We further recommend an upward review of the VAT component to the NHIS fund, special NHIS tax “Sin Tax” to be charged on luxurious goods and services such as cigarettes and alcohol and communication “Talk” tax in the telecommunication sector to increase the revenue base of the scheme.

## Abbreviations

COHEiSION Project: Client-oriented health insurance system in Ghana project
GLSS: Ghana Living Standards Survey
GSS: Ghana Statistical Service
LEAP: Livelihood Empowerment Against Poverty
NHIA: National Health Insurance Authority
NHIF: National health insurance fund
NHIS: National Health Insurance Scheme
NWO: Netherlands Organization for Scientific Research
SSNIT: Social Security and National Insurance Trust
VAT: Value Added Tax
WOTRO: Science for Global Development
